# Left Dorsolateral Prefrontal Cortex Glx/tCr Predicts Efficacy of High Frequency 4- to 6-Week rTMS Treatment and Is Associated With Symptom Improvement in Adults With Major Depressive Disorder: Findings From a Pilot Study

**DOI:** 10.3389/fpsyt.2021.665347

**Published:** 2021-12-03

**Authors:** Pallab Bhattacharyya, Amit Anand, Jian Lin, Murat Altinay

**Affiliations:** ^1^Cleveland Clinic, Imaging Institute, Cleveland, OH, United States; ^2^Department of Radiology, Cleveland Clinic Lerner College of Medicine, Cleveland, OH, United States; ^3^Cleveland Clinic, Neurological Institute, Cleveland, OH, United States

**Keywords:** repetitive transcranial magnetic stimulation (rTMS), major depressive disorder (MDD), magnetic resonance spectroscopy (MRS), glutamate, gamma aminobutyric acid (GABA)

## Abstract

About 20–40% of estimated 121 million patients with major depressive disorder (MDD) are not adequately responsive to medication treatment. Repetitive transcranial magnetic stimulation (rTMS), a non-invasive, non-convulsive neuromodulation/neurostimulation method, has gained popularity in treatment of MDD. Because of the high cost involved in rTMS therapy, ability to predict the therapy effectiveness is both clinically and cost wise significant. This study seeks an imaging biomarker to predict efficacy of rTMS treatment using a standard high frequency 10-Hz 4- to 6-week protocol in adult population. Given the significance of excitatory and inhibitory neurotransmitters glutamate (Glu) and gamma aminobutyric acid (GABA) in the pathophysiology of MDD, and the involvement of the site of rTMS application, left dorsolateral prefrontal cortex (lDLPFC), in MDD, we explored lDLPFC Glx (Glu + glutamine) and GABA levels, measured by single voxel magnetic resonance spectroscopy (MRS) with total creatine (tCr; sum of creatine and phosphocreatine) as reference, as possible biomarkers of rTMS response prediction. Mescher-Garwood point-resolved spectroscopy (MEGA-PRESS) MRS data from 7 patients (40–74 y) were used in the study; 6 of these patients were scanned before and after 6 weeks of rTMS therapy. Findings from this study show inverse correlation between pretreatment lDLPFC Glx/tCr and (i) posttreatment depression score and (ii) change in depression score, suggesting higher Glx/tCr as a predictor of treatment efficacy. In addition association was observed between changes in depression scores and changes in Glx/tCr ratio. The preliminary findings did not show any such association between GABA/tCr and depression score.

## Introduction

Major depressive disorder (MDD), which has a lifetime prevalence of 15% (1), does not respond adequately to medication treatment in ~20–40% of affected patients (2), and these patients have higher morbidity and mortality than those with disease that responds to medication (3, 4). Although electrical stimulation techniques such as electroconvulsive therapy (5–7), vagus nerve stimulation (8–10), and deep brain stimulation (11–13) are suitable for medication-resistant MDD, they are invasive in nature. Repetitive transcranial magnetic stimulation (rTMS), on the other hand, is a non-invasive, non-convulsive neuromodulation/neurostimulation method that has gained popularity for the treatment of MDD that is not responsive to medication (14–37). In particular, high-frequency (>5 Hz) rTMS applied to the left dorsolateral prefrontal cortex (lDLPFC) has been found to significantly decrease Hamilton Depression Rating Scale (HAM-D) scores in patients with medication-resistant MDD (38, 39). Standard and most optimal rTMS therapies are administered at a frequency of 10 Hz (40, 41) over 4–6 weeks (40).

In patients with MDD, rTMS has been reported to change the balance of excitation and inhibition in cortical networks (42, 43), and the antidepressant effect from rTMS has been attributed in part on modulation of the major excitatory neurotransmitter glutamate (Glu) and the major inhibitory neurotransmitter gamma aminobutyric acid (GABA) (44). Multiple studies have documented the involvement of these neurotransmitters in the pathophysiology of MDD (45–51). Changes in cortical Glu or Glx (Glu + glutamine) and GABA levels in patients with MDD have been investigated using *in vivo* magnetic resonance spectroscopy (MRS). In spite of some differences in acquisition (e.g., Mescher-Garwood point-resolved spectroscopy [MEGA-PRESS] vs. short echo time [TE] PRESS), analysis, and quantification methodologies (e.g., absolute levels vs. ratios), these studies have demonstrated a reduction in cortical Glu or Glx and GABA levels associated with MDD (45, 48–50, 52). More specifically, reduced PFC Glx level in patients with MDD has been reported in several studies (45, 53, 54). Researchers have suggested that dysfunction of the glutamatergic system and malfunction in Glu metabolism are contributing factors to the neurobiology and pathophysiology of MDD (55, 56), and the efficacy of glutamatergic agents (glutamatergic targets/receptors such as ketamine, mamantine, riluzole, dextromethorphan, AZD6765 etc.) for the treatment of MDD has been reported (56, 57). Studies have also shown that reduced cortical GABA level is associated with dysfunctional GABAergic interneurons and GABA_A_ receptors; affected GABAergic transmission has been proposed as a mechanism of MDD (58–60). rTMS studies have shown deficits in cortical inhibition in adults with MDD (61, 62); while in children and adolescents increased excitatory cortical facilitation with unchanged cortical inhibition was observed (63).

Multiple *in vivo* studies of Glu and GABA modulation after rTMS in patients with MDD have been performed (58, 64–66). In one study using MEGA-PRESS, the medial prefrontal cortex (MPFC) Glu level was unchanged but the GABA level was elevated after 25 sessions of 10-Hz rTMS therapy applied at the lDLPFC (58). In another study using PRESS and involving 10 sessions of 20-Hz rTMS, an increase in Glu level was seen in the DLPFC, with no changes seen in the anterior cingulate cortex (64). In a short TE PRESS study of young adults treated with 10-Hz rTMS for 15 days, the lDLPFC Glu level was increased in responders but reduced in non-responders (65). Another study using MEGA-PRESS demonstrated an increase in DLPFC GABA level after 6 weeks of 10-Hz rTMS therapy (67).

The prefrontal cortex has been shown to be important in the pathogenesis of MDD (68, 69), and decreased activation of the cortical areas of the mood-regulating circuit has also been reported in patients with MDD (70, 71). More specifically, several studies have shown abnormalities in the DLPFC in patients with MDD (72–76), with affected patients demonstrating reduced levels of GABA and Glx (Glu + glutamine [Gln]) in the DLPFC (45, 77, 78). Lower metabolic activity in the DLPFC (79) as well as lower functional connectivity within the cognitive control network (80), a network that contains the DLPFC, has been reported in depression. In addition MDD is associated with reduced prefrontal cortex gray matter volume, cell counts and glucose metabolism (81). These abnormal (mostly left) prefrontal cortex activities in MDD therefore make the DLPFC a logical and popular rTMS target (73, 81–83).

Differences in Glu levels between responders and non-responders to antidepressants (84) and rTMS therapy (64, 65) suggest that Glu level is a predictor of therapy outcomes in MDD. More specifically, studies have demonstrated that responders to rTMS therapy have lower baseline DLPFC Glu levels than non-responders (64, 65), suggesting that baseline Glu level could be a predictor of response to rTMS therapy. However, most of these studies included only young adults or were carried out over a different period of time than the standard and optimal 4- to 6-week period (40). Thus, additional research is needed to establish an imaging biomarker that can be used to predict the success of rTMS treatment using a standard 10-Hz (40, 41) 4- to 6-week protocol in the adult population. Identifying such biomarker is significant from out of pocket patient expense also, since rTMS therapy is quite costly (can range from ~6,000 to ~$15,000 for 30 sessions in the USA depending on the location, center, applicable discounts and insurance coverage) and is often not covered by insurance.

In this longitudinal study, we measured Glx/tCr and GABA/tCr at the lDLPFC, the site of rTMS application, to determine whether the baseline measures of these could be used to predict outcomes after 6 weeks of 10-Hz rTMS therapy. To this end, we assessed the association between these baseline ratios (Glx/tCr and GABA/tCr) and change in 17-item Hamilton Depression (HAM-D) score after rTMS, as well as the association between the baseline ratios and posttreatment HAM-D score. In addition, we evaluated the Glx/tCr and GABA/tCr ratios to track recovery after rTMS therapy, i.e., we assessed the association between the changes of these ratios and HAM-D scores in response to rTMS therapy.

## Materials and Methods

The study was performed following an IRB-approved protocol. All patients provided written informed consent. We initially enrolled 12 patients (4 men; mean age, 53 y ± 15 y; range, 23–74 y) who had an HAM-D score >15 and who met the DSM-IV-TR (85) criteria for MDD inadequately responsive to at least one antidepressant despite treatment with an adequate dosage for at least 8 weeks (the indication for rTMS approved by the Food and Drug Administration). Patients were recruited from Center of Behavioral Health outpatient psychiatry clinic for mood disorders at our center. Two of these patients did not complete the study, undergoing only 1 MR imaging session and 3 patients had excessive motion during the pretreatment scan; thus, the final analysis consisted of 7 patients.

Of the 7 subjects included in the final analysis, 6 subjects were on antidepressants in combination with low dose 2nd generation neuroleptics (*n* = 4), mood stabilizers (*n* = 2), stimulants (*n* = 2) and other augmentation agents (*n* = 2). Low dose anti-anxiety medications were allowed per inclusion criteria (*n* = 4). Patients were asked to remain on the same dosages on all of the medications during the course of the rTMS treatment. No new medications and/or other non-medication treatment modalities were started at least 1 month before or during the acute rTMS series.

### rTMS Protocol

rTMS was performed using a MagPro R-30 magnetic stimulator (MagVenture, Farum, Denmark) with “cool B-65” magnetic coil, a device that has been used effectively in previous studies (14, 86, 87). Each patient underwent rTMS therapy sessions 5 times per week for a total of 6 weeks (total of 30 rTMS sessions); we selected a duration of 6 weeks because previous studies have used 4–6 weeks of treatment to testy for rTMS effectiveness (14, 88, 89). Each session lasted ~40 min and used the following parameters: frequency, 10 Hz; power, 120% of the motor threshold (i.e., minimum amount of energy needed to trigger thumb movement); duration of stimulus, 4 s; intertrain interval, 26 s; number of pulses per train, 75; and total number of pulses, 3,000. In order to locate the lDLPFC, first the left motor strip controlling the movements of the right thumb was located. The coil was then advanced 5 cm on to the anterior of the motor strip to target the lDLPFC. An experienced staff psychiatrist (MA) administered the rTMS and also performed HAM-D assessment at baseline and every 2 weeks.

### MR Imaging

MR scans were performed on a Siemens 3T Prisma scanner (Erlangen, Germany) using a 20-channel coil head/neck coil. Each patient was scanned within 1 week before starting rTMS therapy (pretreatment scan) and within 1 week after the end of 6 weeks of therapy (posttreatment scan).

**Figure 1:**
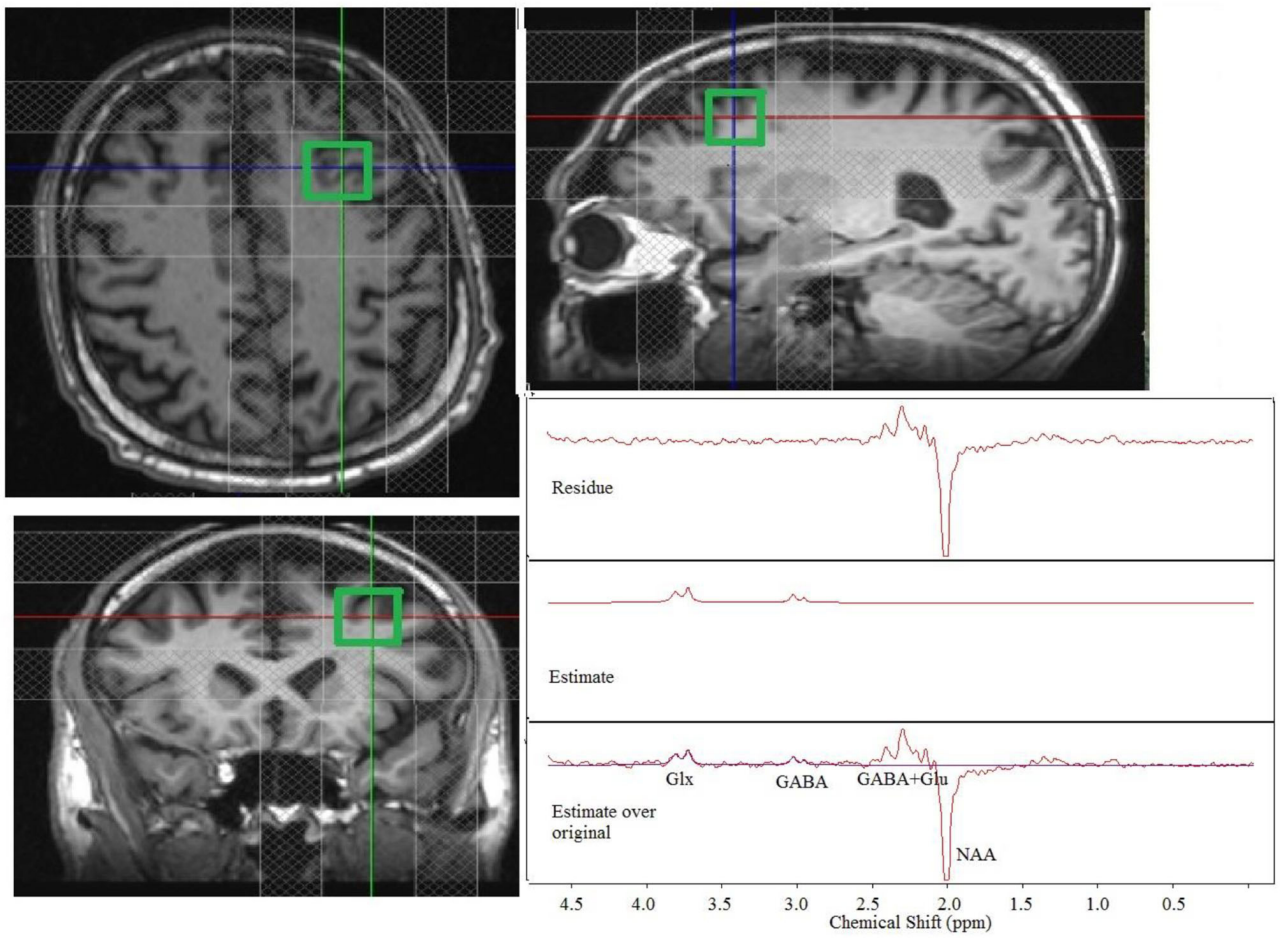
Placement of a 2 × 2 × 2 cm^3^ dorsolateral prefrontal cortex voxel with the outer volume suppression bands and representative single-patient MEGA-PRESS edited spectra (original, estimate and residue). Glx, glutamate + glutamine; GABA, gamma aminobutyric acid; Glu, glutamate.

Each MR session consisted of the following scans: (1) Localizer scan to obtain scout images: scan time, 9 s; (2) Gradient recalled echo scan for field-mapping: 32 axial slices; thickness, 4 mm; field of view (FOV), 256 mm × 256 mm; dual echo times (TE1/TE2)/repetition time (TR)/flip angle (FA), 4.89 ms/7.35 ms/388 ms/60°; matrix, 64 × 64; bandwidth, 260 Hz; scan time, 36 s; (3) T1-weighted anatomical magnetization prepared rapid acquisition gradient echo (MPRAGE) scan: 120 axial slices; thickness, 1.2 mm; FOV, 256 mm × 256 mm; inversion time/TE/TR/FA, 1,900 ms/1.71 ms/900 ms/8°; matrix, 256 × 128; bandwidth, 62 kHz; scan time, 4 min 5 s; and (4) Mescher-Garwood point-resolved spectroscopy (MEGA-PRESS) scan for GABA and Glx measurement of a 2 × 2 × 2 cm^3^ voxel in the lDLPFC: TR, 2,700 ms; TE, 68 ms; frequency-selective 180° pulses at 1.9 (ON-resonance) and 1.5 ppm (OFF-resonance, to minimize macromolecule contamination of GABA); minimum achievable frequency selective pulse bandwidth (~44 Hz); number of averages, 128 per condition (ON-/OFF-resonance); weak water suppression (to use residual water fluctuation to assess patient motion); scan time, 10 min 53 s. A trained technologist ensured that the lDLPFC voxel locations (Figure 1) were closely matched between the pretreatment and posttreatment sessions. The patients bit onto a bite-bar during all scans to reduce head motion. For all spectroscopy scans, shimming was performed using the FASTESTMAP shimming routine (90).

### MRS Data Analysis

Postprocessing of MRS data was performed using the MRUI software package (91) following the method described by Bhattacharyya et al. (92). Postprocessing consisted of zero-order phase correction and frequency shift correction of the individual subspectra using residual water as a reference, averaging the individually phase- and frequency-corrected spectra, residual water suppression with Hankel-Lanczos squares singular value decomposition (HLSVD) filter (93), apodization by a 5-Hz Gaussian filter, and zero filling The OFF-resonance spectrum was subtracted from the ON-resonance spectrum to obtain the final edited spectrum. Motion was identified retrospectively using residual water signal fluctuation as an indicator (92).

Next the ~3.75-ppm Glx and 3.01-ppm GABA peaks from the edited spectrum were fitted as double Gaussian peaks using the AMARES algorithm (94) with zero-order phase correction. The 3.04-ppm creatine (tCr) peak was fitted similarly from the OFF-resonance spectrum. Glx/tCr and GABA/tCr levels were obtained from I_Glx_/I_tCr_ and I_GABA_/I_tCr_, respectively, where I_Glx_, I_GABA_, and I_tCr_ represent areas of the Glx, GABA, and tCr fits, respectively. Edited spectral fitting was done by including the ~2.3 ppm GABA+Glu and inverted NAA peaks as well, but that did not have any effect on I_Glx_ or I_GABA_.

### Statistical Analysis

Percent (%) changes in HAM-D score and Glx/Cr were determined using the expressions


$$\frac{\left(\text{Posttreatment HAMD}\right)-\left(\text{Pretreatment HAMD}\right)}{\text{Pretreatment HAMD}}\times \text{100}$$

and


(1) $$\frac{\left(\text{Posttreatment Glx/Cr}\right)-\left(\text{Pretreatment Glx/Cr}\right)}{\text{Pretreatment Glx/Cr}}\times \text{100}$$

respectively. Non-parametric Wilcoxon signed rank test was between pre- and posttreatment HAM-D scores. Spearman correlation coefficient was used to characterize the association between (1) pretreatment DLPFC Glx/tCr (and GABA/tCr) ratios and changes in HAM-D scores (from pretreatment to 6 weeks posttreatment) and (2) changes in DLPFC Glx/tCr (and GABA/tCr) ratios and changes in HAM-D scores.

## Results

Some MRS datasets had to be discarded because of excessive motion. A total of 3 patients had pretreatment scans that could not be used because of excessive motion, and 1 of these patients also had a posttreatment scan that could not be used because of excessive motion. Thus, 7 motion-free pretreatment scans and 6 motion-free posttreatment scans were used for analysis. A Representative single-patient edited spectra (original, estimate and residual spectra) at the lDLPFC are shown in Figure 1.

From Wilcoxon signed rank test, significant decrease in HAM-D scores was observed for the 10 patients who completed the study (pretreatment score, 20 ± 3; posttreatment score, 8 ± 6; *p* = 0.006). Of the 7 patients (age: 59 ± 13 y) with motion-free pretreatment scans the pretreatment and posttreatment HAM-D scores were 21 ± 3 and 11 ± 8, respectively (*p* = 0.016), while the pretreatment and posttreatment HAM-D scores for the 6 patients (age: 59 ± 13 y) with both motion-free scans were 20 ± 3 and 10 ± 8, respectively (*p* = 0.031).

**Figure 2:**
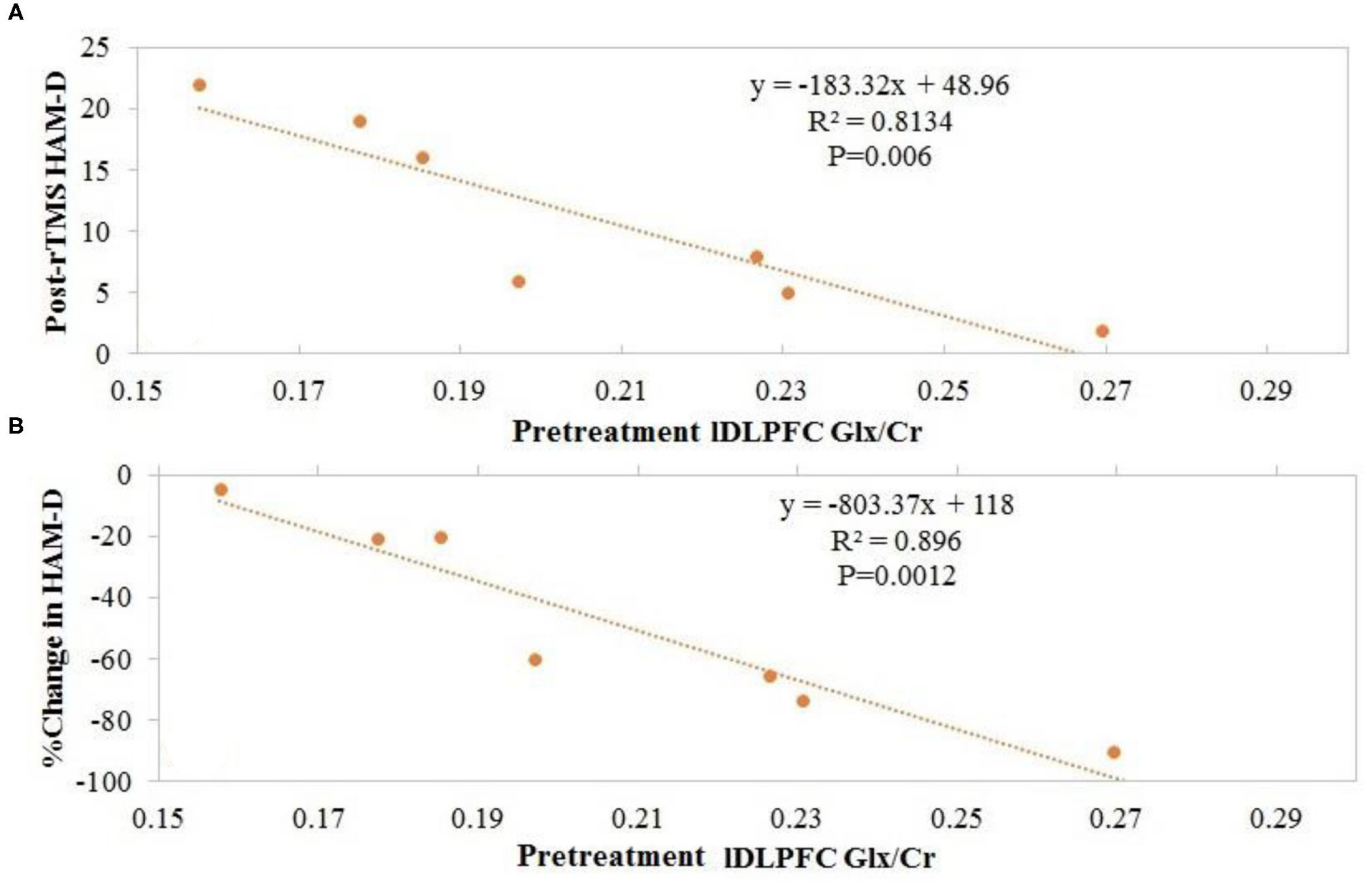
Patients with higher pretreatment glutamate + glutamine (Glx)/total creatine (tCr) at the left dorsolateral prefrontal cortex (lDLPFC) demonstrated **(A)** lower posttreatment Hamilton Depression Rating Scale (HAM-D) scores and **(B)** greater change in HAM-D scores after repetitive transcranial magnetic stimulation (rTMS).

**Figure 3:**
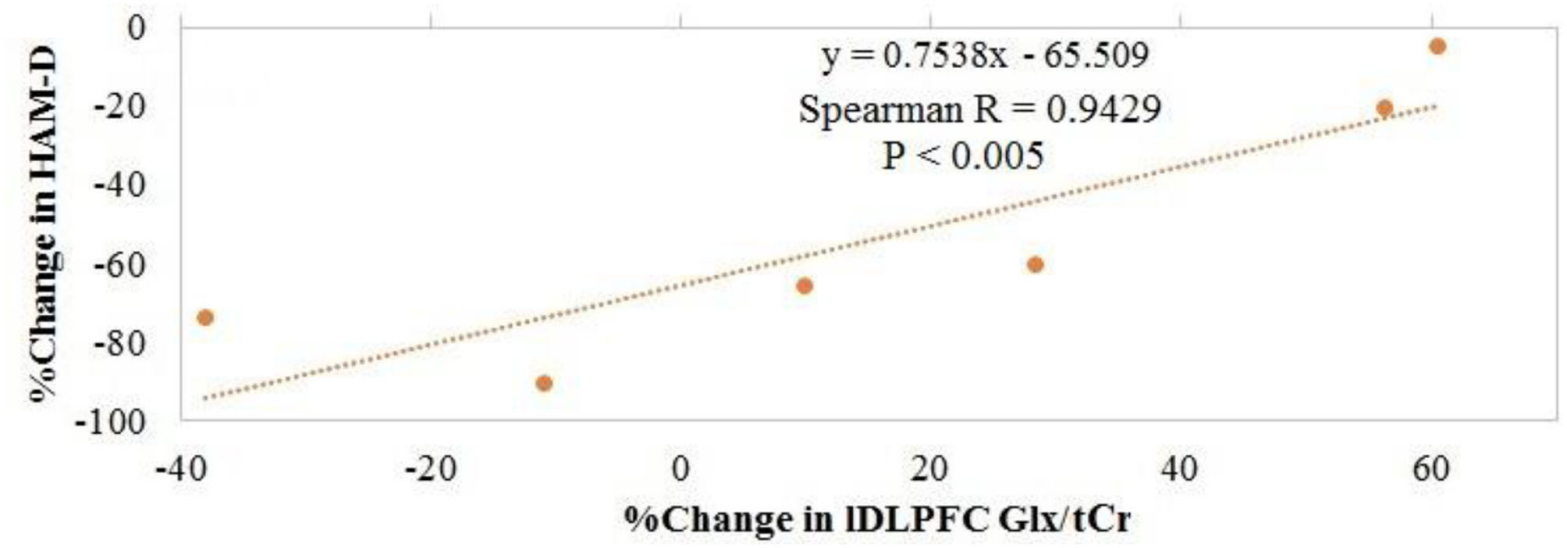
Association between change in glutamate + glutamine (Glx)/total creatine (tCr) in the left dorsolateral prefrontal cortex and change in Hamilton Depression Rating Scale (HAM-D) score.

Overall, no significant changes in Glx/tCr or GABA/tCr were observed as a result of rTMS therapy (Table 1). Inverse Spearman correlations were observed between (1) posttreatment HAM-D score and pretreatment lDLPFC Glx/tCr (*n* = 7; *p* < 0.0005) and (2) change in HAM-D score and pretreatment lDLPFC Glx/tCr (*n* = 7; *p* = 0.001; Figures 2A,B). No such significant correlations were observed between (1) posttreatment HAM-D score and pretreatment GABA/tCr (*n* = 7; *p* = 0.66) and (2) change in HAM-D score and pretreatment lDLPFC GABA/tCr (*n* = 7; *p* = 0.39). A significant correlation was observed between change in HAM-D score and change in Glx/tCr in the lDLPFC (*n* = 6; *p* = 0.02; Figure 3); no such association was observed between change in HAM-D score and change in GABA/tCr (*n* = 6; *p* = 0.45).

**Table 1:** Depression ratings, Glx and GABA levels before and after rTMS treatment.

**Variable**	**Pretreatment value**	**Posttreatment value**	***p***
HAM-D score (*n* = 10)	20 ± 3	11 ± 7	0.0007
Glx/tCr (*n* = 6)	0.21 ± 0.04	0.24 ± 0.05	0.21
GABA/tCr (*n* = 6)	0.11 ± 0.02	0.13 ± 0.06	0.20

*Data are presented as mean ± SD. HAM-D, Hamilton Depression Rating Scale; Glx, glutamate + glutamine; tCr, total creatine; GABA, gamma aminobutyric acid*.

It should be pointed out that HAM-D scores were obtained every 2 weeks and similar analyses were run using the 4-week HAM-D scores. Significant decrease in HAM-D scores were observed in 7 patients with motion-free pretreatment scans (4-week HAM-D score = 10 ± 7, *p* = 0.022). Similar to 6-week data, inverse Spearman correlations were seen between (1) 4-week HAM-D score and pretreatment lDLPFC Glx/tCr (*n* = 7; *p* < 0.0005) and (2) change in HAM-D score in 4 weeks and pretreatment lDLPFC Glx/tCr (*n* = 7; *p* < 0.0005).

## Discussion

In this study, patients treated with 6 weeks of 10-Hz rTMS targeting the lDLPFC demonstrated a decrease in HAM-D score; however, no overall changes in Glx/tCr or GABA/tCr ratios (averaged over 6 patients) were observed. One previous study reported no change in the MPFC Glu level after 25 sessions of 10-Hz rTMS therapy (58); however, an increase in MPFC GABA+ (GABA+macromolecule) level was observed. Although this previous study had a higher number of patients (*n* = 23) than the current study, the region of interest (MPFC) was different from the site of rTMS application (lDLPFC), which was evaluated in the current study.

In this study, patients with higher pretreatment Glx/tCr had lower posttreatment HAM-D scores and larger reductions in HAM-D score after 6 (as well as 4) weeks of rTMS. This finding, albeit from a small sample, is promising and suggests that lDLPFC Glu level may be a predictor of 4- to 6-week rTMS outcome. It should be noted that a higher baseline lDLPFC Glu level has also been previously reported in responders to antidepressant therapy (84), indicating that the predictive power of lDLPFC Glu level may not be limited to rTMS. On the other hand, a lower baseline Glu level has also been reported in youth responders to 3 weeks of rTMS (65), which is the opposite of what we observed in the current study. We speculate that this difference results from the difference in age groups between the studies. Cerebral Glu level has been reported to decrease with age (95); hence, in the older patient population as in this study (40–74 y for the patients who completed the study and had motion-free pretreatment scans), a higher pretreatment Glu level may favor the therapeutic action of rTMS. There was no overall change in Glx/tCr after rTMS. Our results indicate that while Glx/tCr in the lDLPFC increased in 4 patients and decreased in 2 patients, a decrease in HAM-D was associated with a lesser increase or larger decrease in Glx/tCr ratio.

Baseline GABA level in this preliminary study was not associated with response to rTMS therapy, and no previous studies have demonstrated evidence of such a relationship. Additionally, no association between baseline prefrontal cortex GABA level and improvement in MDD was observed in a study assessing ketamine infusion therapy (96). It is likely, therefore, that the baseline GABA level does not predict recovery from MDD irrespective of the treatment regimen.

Test-retest reliability of Glx/Cr and GABA/Cr measurements of a 2 × 2 × 2 cm^3^ voxel in the lDLPFC using MEGA-PRESS sequence was evaluated independently in our center as described in the Supplementary Material. The test-retest variability (9.2%) of Glx/Cr is less than that observed in response to rTMS treatment, while the corresponding GABA/Cr changes for two subjects were less than the variability (16.6%).

While a direct connection between Glx and excitatory neurotransmission is not obvious, it should be noted that Glx measured with the MEGA-PRESS sequence (97, 98) used in this study has been reported to contain mostly Glu with little or no Gln and is therefore considered a good measure of Glu (58, 99–101). Based upon those reports, we speculate that much of our findings pertain to the involvement of excitatory Glu in rTMS therapy. However, we do recognize that there could be a small contribution of Gln in the Glx peak.

A higher lDLPFC Cr in MDD than in healthy controls has been reported (102). In this study, Glx/tCr and GABA/tCr ratios are reported, with areas of the respective resonances in the MEGA-PRESS edited spectra normalized to tCr area from OFF-resonance spectra. For technical reasons, water-unsuppressed MEGA-PRESS scans were not incorporated in the protocol at the beginning of the study; however, those scans were added after the scans of the first 2 patients were completed. Normalizing Glx and GABA to tCr is a well-established method (103–105), and we validated this in our dataset by correlating Glx and GABA normalized to unsuppressed water with Glx/tCr and GABA/tCr from all studies with unsuppressed water acquisition (i.e., from both MRI sessions for patients who completed the study and from pretreatment visits for patients who dropped out after 1 MRI session). The 2 metrics were correlated (*p* = 0.001 for Glx and 0.0001 for GABA), which validated usage of ratio with respect to tCr for this patient population.

Lack of any observed association of GABA in this preliminary study should be treated with caution. It is possible that the main reason for the lack of any significant changes in GABA/tCr ratios or any correlations therewith in this study is the lack of statistical power with 6 subjects. Spectral fitting error was ~30% worse in GABA than in case of Glx, which would result in lower sensitivity of detecting GABA association. Signal to noise ratio and fitting error can be improved with GABA+ acquisition (103), but our choice of macromolecule-minimized GABA accounts for any inter-subject macromolecule level differences (106).

This study had some limitations, including its small sample size. However, a power analysis with *n* = 7 yielded power of 0.95 and 0.85, respectively, for the inverse correlation observed between pretreatment lDLPFC Glx/tCr and change in HAM-D and posttreatment HAM-D, respectively. In addition, with *n* = 6 the study had power of 0.80 to detect 18% change in Glx/tCr ratio. The study also did not include a sham treatment population, which may raise questions regarding glutamatergic involvement in the improvement of MDD as an effect of rTMS. Use of the standard 5-cm rule for rTMS target selection is another limitation, as use of neuronavigation instead has been shown to ensure more reliable, precise, and consistent targeting of the desired brain region (107). Finally, low doses of neuroleptics, benzodiazepine (not more than 1–2 mg), and mood stabilizers were allowed in the study; we did not assess the potential effects of these medications on the study findings. However, for all medications a fixed dose for 4 weeks (6 weeks for benzodiazepines) before rTMS with no change in medication during rTMS treatment was followed as part of the study protocol to minimize any medication effect to the observations reported in this study. We have covered a wide range of age in this study. We hypothesize that while the baseline metabolite levels may be varying due to age and drug regimen, the change in those levels in 6 weeks (study period) will be due to rTMS therapy.

## Conclusion

This study found that the most commonly used rTMS protocol (10 Hz, 4–6 weeks, lDLPFC target) did not significantly change lDLPFC Glx/tCr or GABA/tCr ratios in adults with MDD. Patients with higher pretreatment lDLPFC Glx/tCr ratio did respond better to rTMS therapy; they had a greater reduction in HAM-D score and a lower posttreatment HAM-D score. These findings suggest that excitatory Glu is associated with recovery from MDD and can potentially be used as a biomarker to predict response to rTMS treatment, whereas no such relationship between inhibitory GABA and MDD/rTMS outcome was observed in this preliminary study. The results of this pilot study should be interpreted with caution because of the small sample size and absence of a sham arm; further studies using larger sample sizes are needed to assess these preliminary results.

## Data Availability Statement

The raw data supporting the conclusions of this article will be made available by the authors, without undue reservation.

## Ethics Statement

The studies involving human participants were reviewed and approved by Institutional Review Board, Cleveland Clinic. The patients/participants provided their written informed consent to participate in this study.

## Author Contributions

PB: MRI study design, planning, funding acquisition, MRI data analysis, and writing—original draft. AA: conceptualization, study design, writing—review, and editing. JL: MRI data processing. MA: patient recruitment and consenting, prescribing and administering rTMS, depression rating, writing—review, and editing. All authors contributed to the article and approved the submitted version.

## Funding

Cleveland Clinic Research Program Committee partially funded this project.

## Conflict of Interest

The authors declare that the research was conducted in the absence of any commercial or financial relationships that could be construed as a potential conflict of interest.

## Publisher's Note

All claims expressed in this article are solely those of the authors and do not necessarily represent those of their affiliated organizations, or those of the publisher, the editors and the reviewers. Any product that may be evaluated in this article, or claim that may be made by its manufacturer, is not guaranteed or endorsed by the publisher.

## References

[1] Tundo A de Filippis R Proietti L. Pharmacologic approaches to treatment resistant depression: evidences and personal experience. World J Psychiatry. (2015) 5:330–41. 10.5498/wjp.v5.i3.330 26425446 PMC4582308

[2] Fava M. Diagnosis and definition of treatment-resistant depression. Biol Psychiatry. (2003) 53:649–59. 10.1016/S0006-3223(03)00231-2 12706951

[3] Russell JM Hawkins K Ozminkowski RJ Orsini L Crown WH Kennedy S. The cost consequences of treatment-resistant depression. J Clin Psychiatry. (2004) 65:341–7. 10.4088/JCP.v65n0309 15096073

[4] Reutfors J Andersson TM Brenner P Brandt L DiBernardo A Li G. Mortality in treatment-resistant unipolar depression: a register-based cohort study in Sweden. J Affect Disord. (2018) 238:674–9. 10.1016/j.jad.2018.06.030 29966932

[5] Kellner C. Review: maintenance antidepressants reduce risk of relapse in the 6 months following ECT in people with major depression. Evid Based Ment Health. (2014) 17:8. 10.1136/eb-2013-101663 24419098 PMC13404676

[6] de Vreede IM Burger H van Vliet IM. Prediction of response to ECT with routinely collected data in major depression. J Affect Disord. (2005) 86:323–7. 10.1016/j.jad.2005.03.008 15935255

[7] Ruedrich SL Chu CC Moore SL. ECT for major depression in a patient with acute brain trauma. Am J Psychiatry. (1983) 140:928–9. 10.1176/ajp.140.7.928 6859320

[8] Muller HH Kornhuber J Maler JM Sperling W. The effects of stimulation parameters on clinical outcomes in patients with vagus nerve stimulation implants with major depression. J Ect. (2013) 29:e40–2. 10.1097/YCT.0b013e318290f7ed 23728236

[9] Conway CR Chibnall JT Gangwani S Mintun MA Price JL Hershey T. Pretreatment cerebral metabolic activity correlates with antidepressant efficacy of vagus nerve stimulation in treatment-resistant major depression: a potential marker for response? J Affect Disord. (2012) 139:283–90. 10.1016/j.jad.2012.02.007 22397889 PMC3598572

[10] Cristancho P Cristancho MA Baltuch GH Thase ME O'Reardon JP. Effectiveness and safety of vagus nerve stimulation for severe treatment-resistant major depression in clinical practice after FDA approval: outcomes at 1 year. J Clin Psychiatry. (2011) 72:1376–82. 10.4088/JCP.09m05888blu 21295002

[11] Artigas F. Deep brain stimulation in major depression: plastic changes of 5-hydroxytryptamine neurons. Biol Psychiatry. (2014) 76:174–5. 10.1016/j.biopsych.2014.05.008 25012045

[12] Schlaepfer TE Bewernick BH Kayser S Hurlemann R Coenen VA. Deep brain stimulation of the human reward system for major depression–rationale, outcomes and outlook. Neuropsychopharmacology. (2014) 39:1303–14. 10.1038/npp.2014.28 24513970 PMC3988559

[13] Schlaepfer TE Bewernick BH. Deep brain stimulation for major depression. Handb Clin Neurol. (2013) 116:235–43. 10.1016/B978-0-444-53497-2.00018-8 24112897

[14] Bakker N Shahab S Giacobbe P Blumberger DM Daskalakis ZJ Kennedy SH. rTMS of the dorsomedial prefrontal cortex for major depression: safety, tolerability, effectiveness, and outcome predictors for 10 Hz versus intermittent theta-burst stimulation. Brain Stimul. (2015) 8:208–15. 10.1016/j.brs.2014.11.002 25465290

[15] De Raedt R Vanderhasselt MA Baeken C. Neurostimulation as an intervention for treatment resistant depression: from research on mechanisms towards targeted neurocognitive strategies. Clin Psychol Rev. (2015) 41:61–9. 10.1016/j.cpr.2014.10.006 25468571

[16] McGirr A Van den Eynde F Tovar-Perdomo S Fleck MP Berlim MT. Effectiveness and acceptability of accelerated repetitive transcranial magnetic stimulation (rTMS) for treatment-resistant major depressive disorder: an open label trial. J Affect Disord. (2015) 173:216–20. 10.1016/j.jad.2014.10.068 25462419

[17] Tortella G Selingardi PM Moreno ML Veronezi BP Brunoni AR. Does non-invasive brain stimulation improve cognition in major depressive disorder? A systematic review. CNS Neurol Disord Drug Targets. (2014) 13:1759–69. 10.2174/1871527313666141130224431 25470400

[18] Chung SW Hoy KE Fitzgerald PB. Theta-burst stimulation: a new form of tms treatment for depression? Depress Anxiety. (2015) 32:182–92. 10.1002/da.22335 25450537

[19] Boldt I Eriks-Hoogland I Brinkhof MW de Bie R Joggi D von Elm E. Non-pharmacological interventions for chronic pain in people with spinal cord injury. Cochrane Database Syst Rev. (2014) 11:CD009177. 10.1002/14651858.CD009177.pub2 25432061 PMC11329868

[20] Gaynes BN Lloyd SW Lux L Gartlehner G Hansen RA Brode S. Repetitive transcranial magnetic stimulation for treatment-resistant depression: a systematic review and meta-analysis. J Clin Psychiatry. (2014) 75:477–89; quiz 89. 10.4088/JCP.13r08815 24922485

[21] Leong K Chan P Grabovac A Wilkins-Ho M Perri M. Changes in mindfulness following repetitive transcranial magnetic stimulation for mood disorders. Can J Psychiatry. (2013) 58:687–91. 10.1177/070674371305801206 24331288

[22] Nakamura M. Therapeutic application of repetitive transcranial magnetic stimulation for major depression. Seishin Shinkeigaku Zasshi. (2012) 114:1231–49. 23367835

[23] Lefaucheur JP Andre-Obadia N Poulet E Devanne H Haffen E Londero A. French guidelines on the use of repetitive transcranial magnetic stimulation (rTMS): safety and therapeutic indications. Neurophysiol Clin. (2011) 41:221–95. 10.1016/j.neucli.2011.10.062 22153574

[24] Foucher JR Luck D Chassagnon S Offerlin-Meyer I Pham BT. What is needed for rTMS to become a treatment?. Encephale. (2007) 33:982–9. 10.1016/j.encep.2007.06.002 18789791

[25] Fregni F Marcolin MA Myczkowski M Amiaz R Hasey G Rumi DO. Predictors of antidepressant response in clinical trials of transcranial magnetic stimulation. Int J Neuropsychopharmacol. (2006) 9:641–54. 10.1017/S1461145705006280 16939662

[26] Lam RW Chan P Wilkins-Ho M Yatham LN. Repetitive transcranial magnetic stimulation for treatment-resistant depression: a systematic review and metaanalysis. Can J Psychiatry. (2008) 53:621–31. 10.1177/070674370805300909 18801225

[27] Pallanti S Bernardi S. Neurobiology of repeated transcranial magnetic stimulation in the treatment of anxiety: a critical review. Int Clin Psychopharmacol. (2009) 24:163–73. 10.1097/YIC.0b013e32832c2639 19455047

[28] Richieri R Adida M Dumas R Fakra E Azorin JM Pringuey D. Affective disorders and repetitive transcranial magnetic stimulation: therapeutic innovations. Encephale. (2010) 36(Suppl. 6):S197–201. 10.1016/S0013-7006(10)70057-9 21237356

[29] Schutter DJ van Honk J. A framework for targeting alternative brain regions with repetitive transcranial magnetic stimulation in the treatment of depression. J Psychiatry Neurosci. (2005) 30:91–7. 15798784 PMC551160

[30] Fitzgerald P. Repetitive transcranial magnetic stimulation and electroconvulsive therapy: complementary or competitive therapeutic options in depression? Australas Psychiatry. (2004) 12:234–8. 10.1080/j.1039-8562.2004.02113.x 15715781

[31] Padberg F Moller HJ. Repetitive transcranial magnetic stimulation: does it have potential in the treatment of depression? CNS Drugs. (2003) 17:383–403. 10.2165/00023210-200317060-00002 12696999

[32] Burt T Lisanby SH Sackeim HA. Neuropsychiatric applications of transcranial magnetic stimulation: a meta analysis. Int J Neuropsychopharmacol. (2002) 5:73–103. 10.1017/S1461145702002791 12057034

[33] McNamara B Ray JL Arthurs OJ Boniface S. Transcranial magnetic stimulation for depression and other psychiatric disorders. Psychol Med. (2001) 31:1141–6. 10.1017/S0033291701004378 11681540

[34] Boutros NN Berman RM Hoffman R Miano AP Campbell D Ilmoniemi R. Electroencephalogram and repetitive transcranial magnetic stimulation. Depress Anxiety. (2000) 12:166–9. 10.1002/1520-6394(2000)12:3<166::AID-DA8>3.0.CO;2-M 11126191

[35] Post RM Kimbrell TA McCann UD Dunn RT Osuch EA Speer AM. Repetitive transcranial magnetic stimulation as a neuropsychiatric tool: present status and future potential. J Ect. (1999) 15:39–59. 10.1097/00124509-199903000-00005 10189618

[36] O'Reardon JP Solvason HB Janicak PG Sampson S Isenberg KE Nahas Z. Efficacy and safety of transcranial magnetic stimulation in the acute treatment of major depression: a multisite randomized controlled trial. Biol Psychiatry. (2007) 62:1208–16. 10.1016/j.biopsych.2007.01.018 17573044

[37] Slotema CW Blom JD Hoek HW Sommer IE. Should we expand the toolbox of psychiatric treatment methods to include Repetitive Transcranial Magnetic Stimulation (rTMS)? A meta-analysis of the efficacy of rTMS in psychiatric disorders. J Clin Psychiatry. (2010) 71:873–84. 10.4088/JCP.08m04872gre 20361902

[38] Brunelin J Poulet E Boeuve C Zeroug-vial H d'Amato T Saoud M. Efficacy of repetitive transcranial magnetic stimulation (rTMS) in major depression: a review. Encephale. (2007) 33:126–34. 10.1016/S0013-7006(07)91542-0 17675907

[39] Rizvi S Khan AM. Use of transcranial magnetic stimulation for depression. Cureus. (2019) 11:e4736. 10.7759/cureus.4736 31355095 PMC6649915

[40] McClintock SM Reti IM Carpenter LL McDonald WM Dubin M Taylor SF. Consensus recommendations for the clinical application of repetitive transcranial magnetic stimulation (rTMS) in the treatment of depression. J Clin Psychiatry. (2018) 79:16cs10905. 10.4088/JCP.16cs10905 28541649 PMC5846193

[41] Philip NS Carpenter SL Ridout SJ Sanchez G Albright SE Tyrka AR. 5Hz Repetitive transcranial magnetic stimulation to left prefrontal cortex for major depression. J Affect Disord. (2015) 186:13–7. 10.1016/j.jad.2014.12.024 26210705 PMC4565741

[42] Fitzgerald PB Fountain S Daskalakis ZJ. A comprehensive review of the effects of rTMS on motor cortical excitability and inhibition. Clin Neurophysiol. (2006) 117:2584–96. 10.1016/j.clinph.2006.06.712 16890483

[43] Lenz M Vlachos A. Releasing the cortical brake by non-invasive electromagnetic stimulation? rTMS induces LTD of GABAergic neurotransmission. Front Neural Circuits. (2016) 10:96. 10.3389/fncir.2016.00096 27965542 PMC5124712

[44] Spronk D Arns M Fitzgerald PB. Repetitive transcranial magnetic stimulation in depression: protocols, mechanisms and new developments. In: Cohen Evans E editors. Neuromodulation and Neurofeedback: Techniques and Applications (2010).

[45] Hasler G van der Veen JW Tumonis T Meyers N Shen J Drevets WC. Reduced prefrontal glutamate/glutamine and gamma-aminobutyric acid levels in major depression determined using proton magnetic resonance spectroscopy. Arch Gen Psychiatry. (2007) 64:193–200. 10.1001/archpsyc.64.2.193 17283286

[46] Hashimoto K. Emerging role of glutamate in the pathophysiology of major depressive disorder. Brain Res Rev. (2009) 61:105–23. 10.1016/j.brainresrev.2009.05.005 19481572

[47] Bernard R Kerman IA Thompson RC Jones EG Bunney WE Barchas JD. Altered expression of glutamate signaling, growth factor, and glia genes in the locus coeruleus of patients with major depression. Mol Psychiatry. (2011) 16:634–46. 10.1038/mp.2010.44 20386568 PMC2927798

[48] Rosenberg DR Macmaster FP Mirza Y Smith JM Easter PC Banerjee SP. Reduced anterior cingulate glutamate in pediatric major depression: a magnetic resonance spectroscopy study. Biol Psychiatry. (2005) 58:700–4. 10.1016/j.biopsych.2005.05.007 16084860

[49] Auer DP Putz B Kraft E Lipinski B Schill J Holsboer F. Reduced glutamate in the anterior cingulate cortex in depression: an *in vivo* proton magnetic resonance spectroscopy study. Biol Psychiatry. (2000) 47:305–13. 10.1016/S0006-3223(99)00159-6 10686265

[50] Chang L Cloak CC Ernst T. Magnetic resonance spectroscopy studies of GABA in neuropsychiatric disorders. J Clin Psychiatry. (2003) 64(Suppl. 3):7–14. 12662128

[51] Lener MS Niciu MJ Ballard ED Park M Park LT Nugent AC. Glutamate and gamma-aminobutyric acid systems in the pathophysiology of major depression and antidepressant response to ketamine. Biol Psychiatry. (2017) 81:886–97. 10.1016/j.biopsych.2016.05.005 27449797 PMC5107161

[52] Rosenberg DR Mirza Y Russell A Tang J Smith JM Banerjee SP. Reduced anterior cingulate glutamatergic concentrations in childhood OCD and major depression versus healthy controls. J Am Acad Child Adolesc Psychiatry. (2004) 43:1146–53. 10.1097/01.chi.0000132812.44664.2d 15322418

[53] Arnone D Mumuni AN Jauhar S Condon B Cavanagh J. Indirect evidence of selective glial involvement in glutamate-based mechanisms of mood regulation in depression: meta-analysis of absolute prefrontal neuro-metabolic concentrations. Eur Neuropsychopharmacol. (2015) 25:1109–17. 10.1016/j.euroneuro.2015.04.016 26028038

[54] Yuksel C Ongur D. Magnetic resonance spectroscopy studies of glutamate-related abnormalities in mood disorders. Biol Psychiatry. (2010) 68:785–94. 10.1016/j.biopsych.2010.06.016 20728076 PMC2955841

[55] Sanacora G Treccani G Popoli M. Towards a glutamate hypothesis of depression: an emerging frontier of neuropsychopharmacology for mood disorders. Neuropharmacology. (2012) 62:63–77. 10.1016/j.neuropharm.2011.07.036 21827775 PMC3205453

[56] Mathews DC Henter ID Zarate CA. Targeting the glutamatergic system to treat major depressive disorder: rationale and progress to date. Drugs. (2012) 72:1313–33. 10.2165/11633130-000000000-00000 22731961 PMC3439647

[57] Jaso BA Niciu MJ Iadarola ND Lally N Richards EM Park M. Therapeutic modulation of glutamate receptors in major depressive disorder. Curr Neuropharmacol. (2017) 15:57–70. 10.2174/1570159X14666160321123221 26997505 PMC5327449

[58] Dubin MJ Mao X Banerjee S Goodman Z Lapidus KA Kang G. Elevated prefrontal cortex GABA in patients with major depressive disorder after TMS treatment measured with proton magnetic resonance spectroscopy. J Psychiatry Neurosci. (2016) 41:E37–45. 10.1503/jpn.150223 26900793 PMC4853214

[59] Fogaca MV Duman RS. Cortical GABAergic dysfunction in stress and depression: new insights for therapeutic interventions. Front Cell Neurosci. (2019) 13:87. 10.3389/fncel.2019.00087 30914923 PMC6422907

[60] Maciag D Hughes J O'Dwyer G Pride Y Stockmeier CA Sanacora G. Reduced density of calbindin immunoreactive GABAergic neurons in the occipital cortex in major depression: relevance to neuroimaging studies. Biol Psychiatry. (2010) 67:465–70. 10.1016/j.biopsych.2009.10.027 20004363 PMC2823848

[61] Bajbouj M Lisanby SH Lang UE Danker-Hopfe H Heuser I Neu P. Evidence for impaired cortical inhibition in patients with unipolar major depression. Biol Psychiatry. (2006) 59:395–400. 10.1016/j.biopsych.2005.07.036 16197927

[62] Levinson AJ Fitzgerald PB Favalli G Blumberger DM Daigle M Daskalakis ZJ. Evidence of cortical inhibitory deficits in major depressive disorder. Biol Psychiatry. (2010) 67:458–64. 10.1016/j.biopsych.2009.09.025 19922906

[63] Croarkin PE Nakonezny PA Husain MM Melton T Buyukdura JS Kennard BD. Evidence for increased glutamatergic cortical facilitation in children and adolescents with major depressive disorder. JAMA Psychiatry. (2013) 70:291–9. 10.1001/2013.jamapsychiatry.24 23303429

[64] Luborzewski A Schubert F Seifert F Danker-Hopfe H Brakemeier EL Schlattmann P. Metabolic alterations in the dorsolateral prefrontal cortex after treatment with high-frequency repetitive transcranial magnetic stimulation in patients with unipolar major depression. J Psychiatr Res. (2007) 41:606–15. 10.1016/j.jpsychires.2006.02.003 16600298

[65] Yang XR Kirton A Wilkes TC Pradhan S Liu I Jaworska N. Glutamate alterations associated with transcranial magnetic stimulation in youth depression: a case series. J ECT. (2014) 30:242–7. 10.1097/YCT.0000000000000094 24820947

[66] Peng Z Zhou C Xue S Bai J Yu S Li X. Mechanism of repetitive transcranial magnetic stimulation for depression. Shanghai Arch Psychiatry. (2018) 30:84–92. 29736128 10.11919/j.issn.1002-0829.217047 PMC5936045

[67] Levitt JG Kalender G O'Neill J Diaz JP Cook IA Ginder N. Dorsolateral prefrontal gamma-aminobutyric acid in patients with treatment-resistant depression after transcranial magnetic stimulation measured with magnetic resonance spectroscopy. J Psychiatry Neurosci. (2019) 44:386–94. 10.1503/jpn.180230 31199104 PMC6821508

[68] Davidson RJ Pizzagalli D Nitschke JB Putnam K. Depression: perspectives from affective neuroscience. Annu Rev Psychol. (2002) 53:545–74. 10.1146/annurev.psych.53.100901.135148 11752496

[69] Drevets WC. Functional neuroimaging studies of depression: the anatomy of melancholia. Annu Rev Med. (1998) 49:341–61. 10.1146/annurev.med.49.1.341 9509268

[70] Anand A Shekhar A. Brain imaging studies in mood and anxiety disorders: special emphasis on the amygdala. Ann N Y Acad Sci. (2003) 985:370–88. 10.1111/j.1749-6632.2003.tb07095.x 12724172

[71] Drevets WC. Neuroimaging and neuropathological studies of depression: implications for the cognitive-emotional features of mood disorders. Curr Opin Neurobiol. (2001) 11:240–9. 10.1016/S0959-4388(00)00203-8 11301246

[72] Koenigs M Grafman J. The functional neuroanatomy of depression: distinct roles for ventromedial and dorsolateral prefrontal cortex. Behav Brain Res. (2009) 201:239–43. 10.1016/j.bbr.2009.03.004 19428640 PMC2680780

[73] Brunoni AR Vanderhasselt MA. Working memory improvement with non-invasive brain stimulation of the dorsolateral prefrontal cortex: a systematic review and meta-analysis. Brain Cogn. (2014) 86:1–9. 10.1016/j.bandc.2014.01.008 24514153

[74] Fitzgerald PB Oxley TJ Laird AR Kulkarni J Egan GF Daskalakis ZJ. An analysis of functional neuroimaging studies of dorsolateral prefrontal cortical activity in depression. Psychiatry Res. (2006) 148:33–45. 10.1016/j.pscychresns.2006.04.006 17029760

[75] Grimm S Beck J Schuepbach D Hell D Boesiger P Bermpohl F. Imbalance between left and right dorsolateral prefrontal cortex in major depression is linked to negative emotional judgment: an fMRI study in severe major depressive disorder. Biol Psychiatry. (2008) 63:369–76. 10.1016/j.biopsych.2007.05.033 17888408

[76] Oh DH Oh D Son H Webster MJ Weickert CS Kim SH. An association between the reduced levels of SLC1A2 and GAD1 in the dorsolateral prefrontal cortex in major depressive disorder: possible involvement of an attenuated RAF/MEK/ERK signaling pathway. J Neural Transm. (2014) 121:783–92. 10.1007/s00702-014-1189-z 24652383

[77] Michael N Gosling M Reutemann M Kersting A Heindel W Arolt V. Metabolic changes after repetitive transcranial magnetic stimulation (rTMS) of the left prefrontal cortex: a sham-controlled proton magnetic resonance spectroscopy (1H MRS) study of healthy brain. Eur J Neurosci. (2003) 17:2462–8. 10.1046/j.1460-9568.2003.02683.x 12814378

[78] Michael N Erfurth A Ohrmann P Arolt V Heindel W Pfleiderer B. Metabolic changes within the left dorsolateral prefrontal cortex occurring with electroconvulsive therapy in patients with treatment resistant unipolar depression. Psychol Med. (2003) 33:1277–84. 10.1017/S0033291703007931 14580081

[79] Drevets WC Price JL Simpson JR Jr. Todd RD Reich T. Subgenual prefrontal cortex abnormalities in mood disorders. Nature. (1997) 386:824–7. 10.1038/386824a0 9126739

[80] Alexopoulos GS Hoptman MJ Kanellopoulos D Murphy CF Lim KO Gunning FM. Functional connectivity in the cognitive control network and the default mode network in late-life depression. J Affect Disord. (2012) 139:56–65. 10.1016/j.jad.2011.12.002 22425432 PMC3340472

[81] Drevets WC Price JL Furey ML. Brain structural and functional abnormalities in mood disorders: implications for neurocircuitry models of depression. Brain Struct Funct. (2008) 213:93–118. 10.1007/s00429-008-0189-x 18704495 PMC2522333

[82] Cummings JL. Frontal-subcortical circuits and human behavior. Arch Neurol. (1993) 50:873–80. 10.1001/archneur.1993.00540080076020 8352676

[83] Mayberg HS. Modulating dysfunctional limbic-cortical circuits in depression: towards development of brain-based algorithms for diagnosis and optimised treatment. Br Med Bull. (2003) 65:193–207. 10.1093/bmb/65.1.193 12697626

[84] Grimm S Luborzewski A Schubert F Merkl A Kronenberg G Colla M. Region-specific glutamate changes in patients with unipolar depression. J Psychiatr Res. (2012) 46:1059–65. 10.1016/j.jpsychires.2012.04.018 22595871

[85] American Psychatric Association. Diagnostic and Statistical Manual of Mental Disorders. 4th Text Rev ed. Washington, DC: American Psychatric Association (2000).

[86] Downar J Geraci J Salomons TV Dunlop K Wheeler S McAndrews MP. Anhedonia and reward-circuit connectivity distinguish nonresponders from responders to dorsomedial prefrontal repetitive transcranial magnetic stimulation in major depression. Biol Psychiatry. (2014) 76:176–85. 10.1016/j.biopsych.2013.10.026 24388670

[87] Salomons TV Dunlop K Kennedy SH Flint A Geraci J Giacobbe P. Resting-state cortico-thalamic-striatal connectivity predicts response to dorsomedial prefrontal rTMS in major depressive disorder. Neuropsychopharmacology. (2014) 39:488–98. 10.1038/npp.2013.222 24150516 PMC3870791

[88] Fitzgerald PB Huntsman S Gunewardene R Kulkarni J Daskalakis ZJ. A randomized trial of low-frequency right-prefrontal-cortex transcranial magnetic stimulation as augmentation in treatment-resistant major depression. Int J Neuropsychopharmacol. (2006) 9:655–66. 10.1017/S1461145706007176 16959055

[89] Januel D Dumortier G Verdon CM Stamatiadis L Saba G Cabaret W. A double-blind sham controlled study of right prefrontal repetitive transcranial magnetic stimulation (rTMS): therapeutic and cognitive effect in medication free unipolar depression during 4 weeks. Prog Neuropsychopharmacol Biol Psychiatry. (2006) 30:126–30. 10.1016/j.pnpbp.2005.08.016 16242826

[90] Gruetter R. Automatic, localized *in vivo* adjustment of all first- and second-order shim coils. Magn Reson Med. (1993) 29:804–11. 10.1002/mrm.1910290613 8350724

[91] Naressi A Couturier C Devos JM Janssen M Mangeat C de Beer R. Java-based graphical user interface for the MRUI quantitation package. Magma. (2001) 12:141–52. 10.1007/BF02668096 11390270

[92] Bhattacharyya PK Phillips MD Stone LA Bermel RA Lowe MJ. Sensorimotor cortex gamma-aminobutyric acid concentration correlates with impaired performance in patients with MS. AJNR Am J Neuroradiol. (2013) 34:1733–9. 10.3174/ajnr.A3483 23493890 PMC7965622

[93] Pijnappel WWF Van den Boogaart A De Beer R Van Ormondt D. SVD-based quantification of magnetic resonance signals. J Magn Reson. (1992) 97:122–4. 10.1016/0022-2364(92)90241-X1449952

[94] Vanhamme L van den Boogaart A Van Huffel S. Improved method for accurate and efficient quantification of MRS data with use of prior knowledge. J Magn Reson. (1997) 129:35–43. 10.1006/jmre.1997.1244 9405214

[95] Chang L Jiang CS Ernst T. Effects of age and sex on brain glutamate and other metabolites. Magn Reson Imaging. (2009) 27:142–5. 10.1016/j.mri.2008.06.002 18687554 PMC3164853

[96] Salvadore G van der Veen JW Zhang Y Marenco S Machado-Vieira R Baumann J. An investigation of amino-acid neurotransmitters as potential predictors of clinical improvement to ketamine in depression. Int J Neuropsychopharmacol. (2012) 15:1063–72. 10.1017/S1461145711001593 22040773 PMC3342437

[97] Mescher M Merkle H Kirsch J Garwood M Gruetter R. Simultaneous *in vivo* spectral editing and water suppression. NMR Biomed. (1998) 11:266–72. 10.1002/(SICI)1099-1492(199810)11:6<266::AID-NBM530>3.0.CO;2-J 9802468

[98] Terpstra M Ugurbil K Gruetter R. Direct *in vivo* measurement of human cerebral GABA concentration using MEGA-editing at 7 Tesla. Magn Reson Med. (2002) 47:1009–12. 10.1002/mrm.10146 11979581

[99] Milak MS Proper CJ Mulhern ST Parter AL Kegeles LS Ogden RT. A pilot *in vivo* proton magnetic resonance spectroscopy study of amino acid neurotransmitter response to ketamine treatment of major depressive disorder. Mol Psychiatry. (2016) 21:320–7. 10.1038/mp.2015.83 26283639 PMC4758914

[100] Cleve M Gussew A Reichenbach JR. *In vivo* detection of acute pain-induced changes of GABA+ and Glx in the human brain by using functional 1H MEGA-PRESS MR spectroscopy. Neuroimage. (2015) 105:67–75. 10.1016/j.neuroimage.2014.10.042 25462698

[101] de la Fuente-Sandoval C Reyes-Madrigal F Mao X Leon-Ortiz P Rodriguez-Mayoral O Solis-Vivanco R. Cortico-striatal GABAergic and glutamatergic dysregulations in subjects at ultra-high risk for psychosis investigated with proton magnetic resonance spectroscopy. Int J Neuropsychopharmacol. (2015) 19:pyv105. 10.1093/ijnp/pyv105 26364273 PMC4815472

[102] Yang XR Langevin LM Jaworska N Kirton A Lebel RM Harris AD. Proton spectroscopy study of the dorsolateral prefrontal cortex in youth with familial depression. Psychiatry Clin Neurosci. (2016) 70:269–77. 10.1111/pcn.12392 27059533

[103] Mikkelsen M Barker PB Bhattacharyya PK Brix MK Buur PF Cecil KM. Big GABA: edited MR spectroscopy at 24 research sites. Neuroimage. (2017) 159:32–45. 10.1016/j.neuroimage.2017.07.021 28716717 PMC5700835

[104] Ramadan S Lin A Stanwell P. Glutamate and glutamine: a review of *in vivo* MRS in the human brain. NMR Biomed. (2013) 26:1630–46. 10.1002/nbm.3045 24123328 PMC3849600

[105] Goto N Yoshimura R Kakeda S Nishimura J Moriya J Hayashi K. Six-month treatment with atypical antipsychotic drugs decreased frontal-lobe levels of glutamate plus glutamine in early-stage first-episode schizophrenia. Neuropsychiatr Dis Treat. (2012) 8:119–22. 10.2147/NDT.S25582 22536067 PMC3333782

[106] Bhattacharyya PK. Macromolecule contamination in GABA editing using MEGA-PRESS should be properly accounted for. Neuroimage. (2014) 84:1111–2. 10.1016/j.neuroimage.2013.08.050 24004693

[107] Ahdab R Ayache SS Brugieres P Goujon C Lefaucheur JP. Comparison of “standard” and “navigated” procedures of TMS coil positioning over motor, premotor and prefrontal targets in patients with chronic pain and depression. Neurophysiol Clin. (2010) 40:27–36. 10.1016/j.neucli.2010.01.001 20230933

