# Supplementary material for: Left Dorsolateral Prefrontal Cortex Glx/tCr Predicts Efficacy of High Frequency 4- to 6-Week rTMS Treatment and Is Associated With Symptom Improvement in Adults With Major Depressive Disorder: Findings From a Pilot Study
Source: Front Psychiatry. 2021 Dec 3;12:665347. doi: 10.3389/fpsyt.2021.665347 (PMC8677827; doi:10.3389/fpsyt.2021.665347)
Supplement: Supplementary file 1 [file Data_Sheet_1.pdf]

Supplementary Material

Test-retest of Glx/tCr and GABA/tCr

The test-retest reproducibility of Glx/tCr and GABA/tCr ratio measurements using MEGA-PRESS at Prisma for a 2 × 2 × 2 cm^3 voxel in the lDLPFC was evaluated independently at our center. Five healthy volunteers were scanned with a MEGA-PRESS sequence following the IRB-approved protocol described in Section 2.2 of the main body of the manuscript. Each volunteer was scanned on 2 different days. The time gap between the 2 sessions was initially 2±2 days; however, 2 volunteers had unacceptable motion in their scans and had to return for rescanning, which increased the gap to 14±15 days.

Data analysis for the test-retest cohort was performed following the method described in Section 2.3 of the main body of the manuscript. The scan from 1 of the volunteers who returned for rescanning was still motion corrupted and had to be discarded. Test-retest intraclass coefficient (ICC) and test-retest variability (TRV, within-volunteer variability) were determined following the procedure described by Parsey at al.(1)

For the 4 volunteers with acceptable data, Glx/tCr ratios at the 2 visits were 0.16±0.04 and 0.17±0.05; GABA/tCr ratios were 0.10±0.03 and 0.09±0.01. The ICC for Glx/tCr and GABA/tCr were 0.91 and 0.80 respectively, while the TRV values were 9.2% for Glx/tCr and 16.6% for GABA/tCr.

1. Parsey RV, Slifstein M, Hwang DR, Abi-Dargham A, Simpson N, Mawlawi O, et al. Validation and reproducibility of measurement of 5-HT1A receptor parameters with [carbonyl-11C]WAY-100635 in humans: comparison of arterial and reference tisssue input functions. *J Cereb Blood Flow Metab*. (2000) 20:1111-33. doi: 10.1097/00004647-200007000-00011.
